# Supplementary material for: A Search for Tick-Associated, Bronnoya-like Virus Spillover into Sheep
Source: Microorganisms. 2023 Jan 13;11(1):209. doi: 10.3390/microorganisms11010209 (PMC9865699; doi:10.3390/microorganisms11010209)
Supplement: Supplementary file 1 [file microorganisms-11-00209-s001.zip › microorganisms-2130273-supplementary.pdf]

microorganisms

MDPI

Article

A Search for Tick-Associated, Bronnoya-like Virus Spillover into Sheep

Bianca Elena Bratuleanu [1,2], Cristian Raileanu [2], Delphine Chrétien [1,3], Pablo Guardado-Calvo [4], Thomas Bigot [1,5], Gheorghe Savuta [2], Sarah Temmam [1,3] and Marc Eloit [1,3,6,*]

1 Pathogen Discovery Laboratory, Institut Pasteur, 75015 Paris, France
2 Regional Center of Advanced Research for Emerging Diseases, Zoonoses and Food Safety (ROVETEMERG), "Ion Ionescu de la Brad", Iasi University of Life Sciences, 700490 Iași, Romania
3 WOAH (OIE) Collaborating Centre for Detection and Identification in Humans of Emerging Animal Pathogens, Institut Pasteur, 75015 Paris, France
4 Department of Virology, Structural Virology Unit, CNRS UMR 3569, Institut Pasteur, Université Paris Cité, 75015 Paris, France
5 Bioinformatics and Biostatistics Hub, Université Paris Cité, Institut Pasteur, 75015 Paris, France
6 Department of Biological and Pharmaceutical Sciences, Alfort National Veterinary School, 94700 Maisons-Alfort, France
* Correspondence: marc.eloit@pasteur.fr

Supplementary Materials

Table S1. List of sequences used in Figure 5.

Viral Species	Viral Family	Accession Number
Wenling frogfish arenavirus 2	*Arenaviridae*	YP_009551605
Salmon pescarenavirus 1		QEG08233
Haartman Institute snake virus		YP_009666122
Old schoolhouse virus 1		YP_010086255
Argentinian mammarenavirus		NP_899217
Lassa mammarenavirus		NP_694872
Wenzhou mammarenavirus		YP_009113209
CAS virus		YP_006590093
ROUT virus		YP_009019197
Wenling crustacean virus 9	*Cruliviridae*	YP_009329879
Plasmopara viticola lesion-associated mycobunyavirales-like virus 8	*Discoviridae*	QJX19788
Perilla mosaic virus	*Fimoviridae*	BDP45122
European mountain ash ringspot-associated virus		YP_003104764
Perch actinovirus	*Hantaviridae*	YP_010088064
Wenling red spikefish hantavirus		YP_010085034
Wenling hagfish virus		YP_010085035
Hantaan orthohantavirus		NP_941982
Imjin virus		YP_009362290
Hainan oriental leaf-toed gecko hantavirus		YP_010085031
Longquan Rhinolophus affinis hantavirus 1		UOX72967
Hangzhou leishbuvirus 1	*Leishbuviridae*	UHK03244
Hubei myriapoda virus 5	*Mypoviridae*	APG79329
Grotenhout virus	*Nairoviridae*	ARB16032
Bandia virus		AMT75383

Dugbe orthonairovirus		NP_690576
Hazara virus		YP_009507850
Leopards Hill virus		YP_009111284
Nairobi sheep disease virus		YP_009361832
Punta Salinas virus		AMT75410
Tamdy virus		UCJ02222
Tofla virus		BAU51655
Beiji nairovirus		UXX19123
Pacific coast tick nairovirus		ARF07704
Pustyn virus		QPD01613
Sulina virus		QRR19155
Crimean-Congo hemorrhagic fever orthonairovirus		UXM19310
South Bay virus		ANT80542
Shayang Spider Virus 1		YP_009300680
Sanxia Water Strider Virus 1		YP_009293594
Xinzhou Spider Virus		AJG39272
Pangolin orthonairovirus		URZ29345
Herbert virus strain F23/CI/2004	*Peribunyaviridae*	YP_009507855
Khurdun virus		AHL27169
Lakamha virus		QEO75951
Abras virus		QLA47005
Bertioga virus		QLA47081
Catu virus		YP_009507870
Madrid virus		QLA46935
Sororoca virus		YP_009666895
Utinga virus		YP_009666923
Pacui virus		YP_009666929
Khasan virus		AII79370
Santarem virus		QLA47032
Tanga virus		QLA47025
Scaphoideus titanus bunya-like virus 1	*Phasmaviridae*	QIJ56910
Ferak virus		YP_009664550
Anopheles triannulatus orthophasmavirus		YP_010086189
Sanxia Water Strider Virus 2		YP_010085075
Wuhan Insect virus 2		YP_009270651
Notori virus		AWA82264
Bhanja virus	*Phenuiviridae*	YP_009141013
Hubei diptera virus 3		YP_009329894
Citrus virus A		UUT43438
Cumuto virus		YP_009664615
Fitzroy Crossing tenui-like virus 1		QLJ83469
Hubei diptera virus 4		YP_009330281
Norway phlebovirus 1		YP_010086240
Laurel Lake virus		YP_009667028
Narangue virus		QHA33858
Guadeloupe mosquito phasivirus		QEM39249
Rift Valley fever virus		YP_003848704
Uukuniemi phlebovirus		AWH61706
Leticia virus		AEL29649

Pidgey virus		AOX47534
Apple rubodvirus 1		UUI77343
Rice grassy stunt tenuivirus		UOX74344
Uukuniemi virus		NP941973
Wenzhou Shrimp Virus 1		AJG39256
Blattodean phenui-related virus OKIAV261		QMP82311
Melon severe mosaic tospovirus	*Tospoviridae*	YP_009346017
Tomato necrotic ringspot virus		QQY96596
Wuhan Millipede Virus 2	*Wupedeviridae*	YP_009666319
Almazovo tick virus	Unclassified *Bunyavirales*	QPB70159
Bronnoya virus isolate CT1		USL85421
Bronnoya virus isolate_NOR/A2/Bronnoya/2014		ASY03250
Ixodes ricinus bunyavirus like virus 1 isolate CT5		USL85429
Ubmeje virus		QKK82912
Athtab bunya-like virus		YP_009553307

**Number of sequences**		*Arenaviridae*	*Cruliviridae*	*Discoviridae*	*Fimoviridae*	*Hantaviridae*	*Leishbuviridae*	*Mypoviridae*	Nairoviridae	**Proposed new family**	*Peribunyaviridae*	*Phasmaviridae*	*Phenuiviridae*	*Tospoviridae*	*Wupedeviridae*
49	*Arenaviridae*	–	0,005	0,007	0,005	0,005	0,006	0,006	0,004	0,005	0,006	0,005	0,005	0,006	0,005
1	*Cruliviridae*	0,557	–	0,009	0,006	0,007	0,008	0,007	0,005	0,006	0,006	0,005	0,006	0,009	0,008
5	*Discoviridae*	0,300	0,637	–	0,008	0,008	0,008	0,009	0,006	0,007	0,008	0,006	0,006	0,007	0,009
10	*Fimoviridae*	0,286	0,562	0,366	–	0,006	0,006	0,006	0,005	0,006	0,005	0,005	0,005	0,006	0,006
36	*Hantaviridae*	0,346	0,644	0,420	0,380	–	0,008	0,008	0,007	0,006	0,005	0,005	0,005	0,007	0,008
1	*Leishbuviridae*	0,555	0,891	0,616	0,615	0,677	–	0,009	0,007	0,006	0,007	0,005	0,005	0,007	0,009
1	*Mypoviridae*	0,522	0,875	0,623	0,611	0,665	0,867	–	0,005	0,006	0,006	0,005	0,005	0,006	0,007
21	Nairoviridae	0,236	0,599	0,342	0,328	0,384	0,587	0,565	–	0,004	0,005	0,005	0,005	0,005	0,004
11	**Proposed new family**	**0,361**	**0,650**	**0,396**	**0,394**	**0,452**	**0,689**	**0,683**	**0,403**	–	0,005	0,004	0,004	0,005	0,004
86	*Peribunyaviridae*	0,296	0,549	0,377	0,302	0,390	0,635	0,613	0,332	**0,382**	–	0,006	0,005	0,007	0,007
13	*Phasmaviridae*	0,211	0,514	0,280	0,252	0,299	0,538	0,529	0,245	**0,323**	0,262	–	0,005	0,006	0,005
98	*Phenuiviridae*	0,221	0,558	0,296	0,292	0,351	0,501	0,544	0,264	**0,361**	0,301	0,217	–	0,007	0,006
24	*Tospoviridae*	0,343	0,625	0,419	0,360	0,446	0,676	0,657	0,377	**0,453**	0,342	0,308	0,344	–	0,007
1	*Wupedeviridae*	0,531	0,870	0,624	0,612	0,660	0,867	0,863	0,540	**0,688**	0,606	0,531	0,536	0,665	–

Figure S1. Estimates of net evolutionary divergence between *Bunyavirales* families. The number of amino-acid differences per site from estimation of net average between *Bunyavirales* families are shown. Standard error estimates (evaluated by 1000 bootstrap replicates) are shown above the diagonal. The analysis involved 357 amino acid sequences. All ambiguous positions were removed for each sequence pair. There were a total of 9817 positions in the final dataset. Evolutionary analyses were conducted in MEGA7.

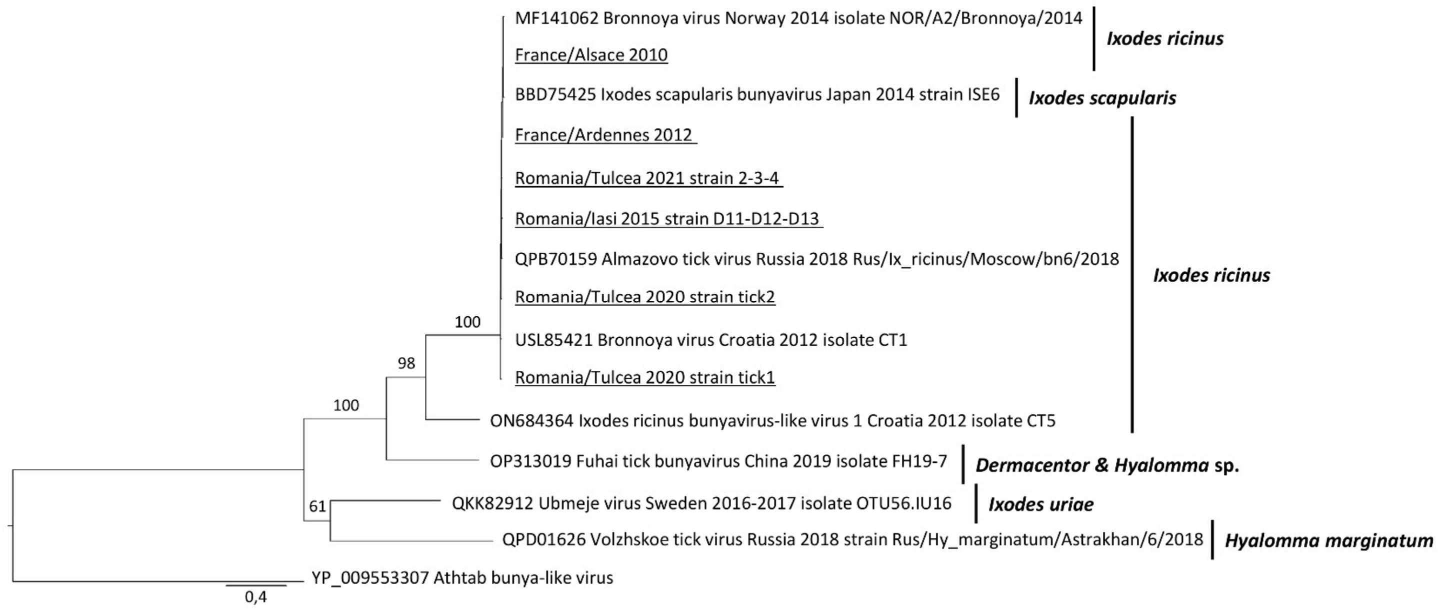

Figure S2. Phylogenetic reconstruction of the partial RNA-dependent RNA polymerase of the proposed new viral family. Sequences generated in this study are underlined.

Disclaimer/Publisher's Note: The statements, opinions and data contained in all publications are solely those of the individual author(s) and contributor(s) and not of MDPI and/or the editor(s). MDPI and/or the editor(s) disclaim responsibility for any injury to people or property resulting from any ideas, methods, instructions or products referred to in the content.
